# “Breaking down the wall” patients` and families` experience of multifamily therapy for young adult women with severe eating disorders

**DOI:** 10.1186/s40337-021-00412-w

**Published:** 2021-04-26

**Authors:** Berit Støre Brinchmann, Sanja Krvavac

**Affiliations:** 1 Regional Centre for Eating Disorders, Nordland Hospital, 8076 Bodø, Norway; 2 The Faculty of Nursing and Health Sciences, Nord University, 8026 Bodø, Norway

**Keywords:** Anorexia nervosa, Bulimia, Eating disorders, Multifamily therapy, (Young) adults, Family

## Abstract

**Background:**

This paper addresses patients` and families` experience of multifamily therapy (MFT) for young adults (18–22) with an eating disorder (ED). EDs are serious illnesses leading to lowered quality of life for the patient and their family. The Regional Centre for Eating Disorders (RESSP) at Nordland Hospital in Bodø, Norway has developed an adjunct psychotherapeutic approach for the treatment of young adult patients with severe EDs. The patient’s family members take part in the multifamily therapy (MFT) group programme.

**Methods:**

The aim of the study was to explore patients` and families` experience of MFT for young adult women with severe EDs. A Grounded Theory (GT) approach was used. Data were collected by field observations in two MFT groups, qualitative group interviews and qualitative individual interviews with patients and their family members. Data were analysed using the constant comparative method. The data analysis consisted of open and selective coding and memo writing.

**Results:**

Two main categories were identified: ‘Connectedness and recognition’ and ‘Opening up and sharing**`.** MFT was described as a space for recognition within which it was possible to speak of things happening in the family with others with similar difficulties. It felt good and freeing, but also painful, to meet others with similar experiences. The participants had in common a considerable loneliness because it is difficult for outsiders to grasp what it is like in a home dominated by an ED. The meeting with other families created an underlying safety. The participants received help to distinguish between realistic and unrealistic concerns and learned about openness and communication in relation to their daughter. Some women with EDs said that MFT was most important for the parents but also had been good for them as things had become better at home.

**Conclusion:**

The participants reported that their family had become better at talking to each other after having been in MFT. As a result, they were able to speak more openly about difficult things and share feelings. This gave rise to increased understanding. The study shows that MFT was found to be valuable and important. Never before had these families had such an opportunity, something so directly tailored to them. MFT for adults can be developed further and used in other groups, such as those concerning other chronic illnesses.

## Plain English summary

Eating Disorders (EDs) are serious illnesses which lead to both sufferers and their families having a much reduced quality of life. The families of adults with a severe ED report a heavy burden of care, isolation and loneliness. The Regional Centre for Eating Disorders (RESSP) at Nordland Hospital in Bodø, Norway has developed an MFT-programme for the treatment of young adults with EDs and their families. Here, we present the findings of a study into how they experienced participation in this therapy. The data were collected by a researcher taking part in the groups as a field observer and who interviewed the group members (the young women and their families). The participants said that they found the MFT to be a place of connectedness and recognition where they learned to be open and share their feelings. They reported that their family had become better at talking to each other, including about difficult matters and feelings. MFT had been valuable and important, and the participants told us that they never before had had something so well-tailored to their needs. We think that MFT for adults will be amenable to further development and applicable to other settings.

## Background

Eating disorders (EDs) are conceptualised as a disturbance in eating patterns or eating-related behaviours, leading to modified absorption or consumption of food [1]. Two prominent types of ED are anorexia nervosa (AN), a severe, enduring disorder characterised by high rates of comorbidity and mortality [2, 3], and bulimia nervosa (BN), a disorder characterised by compensatory behaviours of overeating followed by vomiting, laxative use and excessive exercise [1] leading to impairment in psychosocial functioning and comorbid psychopathology [4].

Family members of patients with an ED can also be affected [5, 6], and their quality of life impaired if their family member is dependent on them [6, 7]. Family members seek help and information from mental health care providers to manage symptoms of EDs and the overwhelming feelings of worry, anxiety, sadness, depression, and helplessness within complex family dynamics. These negative feelings may result in sustained and limited interaction hindering open communication [8]. Families also develop patterns of organisation around the ED that can lead to maintenance of the disorder and may impact the patterns of communication [8]. Family members might often require professional help and support in order to regulate their emotional reactions related to the disorder [9]. It is common to include family members in the MFT-process because the family perspective is crucial to the treatment outcome [10].

MFT is a well-established adjunct psychotherapeutic treatment [11–17]. MFT, as an intensive psychotherapeutic treatment, is based on sharing the experience of the family with other families with similar experiences. Because of the treatment’s intensive aspect, Asen and Scholtz [18] characterise it as a “hothouse effect” contributing to reciprocal learning among family members, leading to both the facilitation of processes such as hope and motivation to change, and alleviation of stigma and perceived isolation.

In the last 15 years, the effect of MFT on AN/BN has been studied mostly in relation to children and adolescents [11–17, 19], with very few studies involving young adults [10, 20–23]. Evidence suggests that its effect may be an improvement in ED symptoms in these patients [11, 12, 19, 20, 24], weight gain [10–12, 19, 20, 24], elevation of mood and self-esteem [24], improved quality of life [12, 21], along with facilitation of motivational drive and elevated insight into the nature of the disorder followed by facilitated communication within the family dynamics [17, 20]. Moreover, patients and family members involved in MFT report significant treatment satisfaction and rates of drop out are low [10–12, 15, 19].

Even though the research evidence looks promising, it should be approached with caution because only one RCT has been conducted [11]. Other studies have not included a control or comparison group [10, 12, 24], or have included only a limited sample size [21].

Furthermore, research suggests that the quality of life of those patients may be limited by stigmatisation from the general public [25]. In turn, fear of stigma in ED patients may lead to isolation and increased reliance on family members for support. Such an expectation from a family member imposes a burden on the whole family, leading to psychological distress, anxiety, depression, stigma, social isolation, and reduced family functioning [26, 27].

Voriadaki and colleagues [17] found that MFT facilitated extensive learning experience and profound insight into the challenges patients and family members experienced, as well as understanding of the nature of the illness directly impacting their quality of life. The same study [17] points out that patients’ and family members’ insight into the disorder contributed to shared externalisation of the disease, leading to the expression of empathy and other emotions. Also, the inclusion of family members was found to be effective in terms of support for patients and patients’ increased motivation, family members’ self-efficacy, resulting in improvement of family dynamics and communication. Moreover, Mehl and colleagues [21] found that integrating families in a multi-group setting leads to a reduction in stigmatisation, loneliness and social isolation, and the sharing of coping strategies along with restructuring of family organisation and relapse prevention. The same study indicated that a safe atmosphere during MFT sessions diverted patients’ and family members’ attention from the disorder, leading to healthier communication and interaction, gaining a positive future perspective with regard to the quality of their lives.

Additionally, it is of crucial importance to understand the experiences and perception of patients and family members regarding MFT intervention [17] because sharing their experience may help improve the MFT service establishment [10], may facilitate insight into the disorder, increase hopefulness and reduce blame and guilt [28]. During MFT, patients experienced parents as “firm”, which led to increased support, especially with eating habits, which improved quality of life in those patients and their family members [17].

Considering that family perspective is central to the MFT and that young adults suffering from AN/BN maintain a closer relationship with their family than is usual at their age, it is important for them and their families to receive a tailored MFT approach, in that they have need of a different service than that offered to children and adolescents. Young adults may show reluctance to include family members in the therapeutic process [20]. Therefore, a tailored approach should consider the nature of the relationship young adults may have with family members, as these young women may be constantly tempted to permanently cut off the contact and get the support they need. Besides, a tailored approach should consider the fact that family members have a deep involvement in the life and the illness of these young adults with severe EDs, which, if ignored, may lead to problematic family communication and social disfunction [10, 29, 30].

At the Regional Centre for Eating Disorders (RESSP) at Nordland Hospital in Bodø, an MFT model as an adjunct psychotherapeutic approach to the treatment of young adult patients with a severe ED has been in continuous development since 2007. The model includes family members, and six to eight families participate, each with a young adult family member suffering from a serious ED. These take an active part in MFT over a 12-month period. This model, developed and delivered at RESSP, is unique and considered, throughout Norway, as being a ground-breaking approach [10, 29–31].

### Study aim

It is important to understand how patients and family members experience MFT. Our study aims to better understand participants’ perceptions of the MFT and its impact on family dynamics and on the quality of family life in participating families. We are looking to both improve and further develop MFT at RESSP. The aim of the study was to explore and describe patients` and families` experience of participating in MFT for young adult women with severe EDs.

### Study setting: MFT at RESSP for eating disordered young adults

The MFT programme for adults with EDs was initially inspired by the practice adopted at the Institute of Psychiatry at the Maudsley Hospital in London [8, 32]. Three therapists took part in an MFT training (for children and young people) over 1.5 years, delivered by Penny Fairbairn and Ivan Eisler. Both have extensive experience of MFT with children and adolescents. Penny Fairborn was asked to supervise during the first stage of the development of MFT at RESSP. The establishment and implementation of the model have been described in detail elsewhere [10, 29–31].

The operative definition of ‘young adult’ in MFT for adults at RESSP is 18–30 years. These young patients must have a primary, ongoing relation with their birth family – that is, have not yet formed a family of their own. They must belong to the catchment area of the regional centre and must have access to a specialist therapist who can provide follow-up care. The family members invited to participate were parents (mother and father), siblings (sisters and brothers) and others particularly close to the patient such as grandparents, partners, boy-girlfriend and, occasionally, close friends. Family members suffering from serious psychological ill-health or suicidal intent or who are suffering substance abuse or dependency are excluded for the sake of a sound group process. The therapists leading the MFT groups are drawn from both the in-patient and out-patient services at RESSP and have widely differing professional backgrounds – psychologists and psychiatrists, social workers, fitness consultants, family and art therapists and psychiatric nurses all contribute – and levels of experience.

MFT in the RESSP model is highly diverse. Because it involves adults, it does not include interventions aimed directly at the eating situation, nor are the patients weighed. Everyone is to eat lunch together on the days the group meets. The aim is not so much to cure the ED, but to support the patients in taking adult responsibility for it, and to encourage and support better communication and interactivity within the family [10, 30]. This is distinct from the MFT conducted with children and adolescents (under 18 s) with EDs where the family has far greater responsibility in ensuring that they eat. Secondary to these aims is that the patients display a reduction in their symptoms, such as a low bodyweight. The MFT incorporates interventions and exercises drawn from many different theoretical models, principally psychodrama, systemic family therapy, collaborative therapy, mentalising-based therapy, mindfulness, multifamily therapy for psychosis and cognitive therapy. A number of the exercises have been developed by the team at RESSP [10, 30].

The MFT at RESSP comprises 6 group gatherings spread over a year, each of either 2- or 3-days’ duration. The main topics for these gatherings are: (1) Establishing the group, (2) Communication and communication styles, (3) Care and patterns of caring, (4) Siblings, (5) ‘Mind the gaps’ concerning transition, and (6) Summing up and ending [10, 33]. MFT uses a variety of group settings - large groups of the patients and family members, and smaller ones (mothers, fathers, siblings, and young women with EDs) - and various interventions including role-play, family mapping and sculpts and other creative work. Both psycho-educational and clinical groupwork sessions focus on relevant and important themes including relationships, communication, conflict management, interactivity, motivation and feelings of guilt [31].

## Method

### Research design

The research design is qualitative and exploratory. This article, which uses a grounded theory (GT) approach [33–36], is part of a wider study of MFT conducted at RESSP at Nordland Hospital in Bodø and is based on fieldwork carried out over 2 years, as well as group- and individual interviews with the patients and family members. An earlier article looked at the practice of the therapists and facilitators involved in the MFT [31].

### Participants

Participants in the study were 12 families, 6 families from each of 2 MFT groups. Twelve young women (18 to 22 years old), 8 with AN and 4 with BN, 12 sets of parents (mothers and fathers 43–60 years old), 9 siblings (8 sisters and 1 brother 16–22 years old), 1 grandmother and 2 partners (of the young women) participated in the 2 MFT groups. The families had in common that each had a daughter suffering a severe ED. Some of the patients were inpatients, but most were being treated in the community.

### Data collection

It is commonplace in GT to make field observations and to hold more in-depth interviews with the participants, both individually and in groups. In GT, ‘everything is data’ [37, 38].

“...the beauty of the method lies in its every-thing-is-data characteristic; that is to say, everything I see, hear, smell, and feel about the target, as well as what I already know from my studies and my life experience, are data. I act as interpreter of the scene I observe, and as such I make it come to life for the reader. I grow it.” [[38] , p.115].

In line with this, neither the number nor the type of interview are pre-planned. Data collection and analysis take place in parallel, and the researcher ‘tests’ the provisional analysis with new interviews with the participants.

Data for the study were collected by means of 180 h of field observation in 2 MFT groups (MFT1 and MFT2), together with both group and individual interviews with the participants: young women and their family members (Table 1). These data were collected by the first author (BSB) between 2015 and 2017. The fieldwork was carried out with BSB present in the MFT groups as a participatory observer, writing contemporaneous field notes whilst taking part in the group activities on much the same basis as other participants. The research was practice-led, in that the researcher participated as an observer on the group’s own terms. The group interviews had to be conducted during breaks, within the time limits this imposed. Nor was it always easy to come into conversation with some of the participants in the MFT – this could be the case particularly with the young women with an ED. This fieldwork formed the basis for the questions in the interviews, using keywords rather than an interview guide. All interviews were recorded and later transcribed. The main questions were: “What is going on in MFT? Tell me about the MFT and what it means for you both as yourself and for your family?” The group interviews (lasting 40–60 min) were all conducted during the final group meeting. Individual interviews (lasting 60–90 min) took place after the interviewees had completed the MFT (see Table 1).

**Table 1:** Overview of participants in the qualitative group interviews and individual qualitative interviews

Group interview 1 (60 min)	5 mothers, 5 fathers	MFT 1
Group interview 2 (40 min)	4 mothers, 2 fathers, one sister, one partner	MFT 2
Group interview 3 (45 min)	4 young women with EDs	MFT 1
Group interview 4 (40 min)	3 young women with EDs	MFT 2
Individual interviews (60–90 min)	6 mothers, 6 fathers, 2 young women with EDs	

‘Theoretical sampling’ [33, 34] normally guides the process of sampling and data collection in GT. This means that the ongoing analysis is, ideally, used by the researcher as a guide to the selection of informants and new areas of interest for further data collection. This may mean a new group or groups of informants who had not been defined or selected in advance. In the case of MFT, where the meetings followed a pre-set schedule, and out of respect for the patients, family members and therapists, BSB elected to be present throughout, even though data collection is normally terminated by having reached saturation point. Group interviews and individual interviews with those – both patients and family members – who had completed the MFT continued until a saturation point was reached (which is to say when no new issues were forthcoming).

### Analysis

GT is a means of deriving theory from empirical data. One considers the patterns from the data and constructs theory, using the constant comparative method. Data collection and data analysis take place in parallel. The data analysis consists of open, selective and theoretical coding and memo writing [33, 34].

The article concerning the therapists [31] represents a GT (theory) about their practice in MFT. In this article, we did not want to develop a theory (GT), in which the findings would be abstracted and presented at a theoretical level, with few quotes. We wanted, rather, to analyse and present the findings in a much more descriptive way, with rich descriptions and quotations and accounts from our informants. GT is not well-suited to conveying a wealth of detail of this kind in the analysis of the data [[39], p.74]. It can, however, be used ‘in part’ [40]. It is possible to report findings at different levels whilst remaining faithful to the principles of GT, so long as the way in which the method has been used and the level at which the analysis has taken place, are made clear [41, 42].

“Glaser often reminds us that GT is good as far as it goes. The first step that you can take with GT is description. I think that GT is a great tool for description … ” [[42] , p. 576].

Strauss and Corbin [43] recommend that one concentrates on, and goes in depth into, one or two categories.

Because we did not want to develop theory, but rather to present our findings descriptively, we restricted ourselves to the first stage of GT analysis – open and selective coding and memo writing. Fieldnotes, and qualitative interviews and group-interviews with the participants in MFT, were transcribed and coded in parallel with further data collection. The transcripts were read line by line and analysed a number of times [33–35]. BSB undertook an initial, provisional analysis of the data. BSB and the second author, SK, then discussed potential main categories. Two research assistants, each with personal experience of MFT as patient and parent respectively, and two MFT therapists also discussed the findings.

### Research ethics

The study conforms to the principles outlined in the Declaration of Helsinki. The research project was approved by the Regional Committee for Medical and Health Research Ethics (REK: reference 2014/1621/REK West). In addition, all participants in the MFT groups received verbal and written information regarding the research project and signed a consent form. They were also informed that they could withdraw from the study at any time. The data were treated confidentially and anonymised.

The trust of the participants was ensured in that BSB was present in the MFT as a participatory observer. She got to know the participants well, and always asked if her presence was acceptable to them, even though both the patients and the family members had agreed to that in advance. They said that BSB had been seen as being part of the group, as if one of the therapists. No-one said that they found the notetaking to be a disturbance. In difficult situations, where there was a lot of emotion, no notes were taken.

## Results

*I was present, as a researcher, in a family group in MFT****.*** *Using Playmobil figures, each family member was to show the others how they experienced the situation in their family. The young woman with an ED placed the ED (a skeleton) in the centre of the table. She put her siblings at the far end of the table, together with a little dog. Mum and Dad were put far apart from each other (they were divorced). “It’s my fault that the family is destroyed,” she said, crying. “Everything would be better if only I were dead.” Her father turned to me and said: “You have to keep hold of that. Now, you have really got to see how things are for us.”* (Fieldnote).

Our analysis shows that the main concern for the participants in MFT for adults with EDs is how to overcome isolation and feeling of loneliness and disconnection in the families. The two main categories explain how participation in MFT help in solving the main concern, through “Connectedness and recognition,” “Opening up and sharing” and “Breaking down the wall”.

### Connectedness and recognition

Connectedness means the state of being joined or linked, a feeling of belonging to or having affinity with a particular group or person. The participants described the MFT as an arena where families could meet, a space for recognition where it was possible to speak of things happening in the family with others with similar difficulties who understand. They spoke of different kinds of understanding and fellowship; in the mothers’ group, the fathers’ group, the young women’s and siblings’ groups and in the large group with everyone present. Many of the group members said that it felt good and freeing, but also painful, to meet other families with similar experiences. What these families had had in common was a considerable loneliness because it is so difficult for outsiders to grasp what it is like in a home dominated by an ED. Here, at last, they had the experience of meeting others who had the same, and who understood how it was for them. A mother expressed this as follows:

*“The first time, the first meeting of the mothers` group, we sat in our group and as soon as we sat down, I began to cry. I was totally unprepared for that because I do not cry easily. It was a shock, but at the same time it was a signal that I had now come to some people who … A strange experience, as I felt I wanted to scream. And I think that it was because I realised that at last there were people who understood what I was talking about … . I understood that there was truth in that you said that you understood what I was talking about – and it was quite liberating – good and bad at the same time.”* (Mother, group interview 1).

The mothers described powerful shared feelings, with a lot of tears and crying, with them all on the same wavelength. They also found relief together, with the other mothers who had had many of the same feelings as them. Several said that they had the experience of starting a sentence, only to find that another of the mothers was able to complete it. During MFT they met others with similar experiences. The recognition that others had gone through the same things, made one feel much less alone with the weight of the world on one’s shoulders.

*“It was fantastic. It was moving. There were family histories. We became just like one big family and everyone opened up.”* (Mother, individual interview 3).

Some of the fathers said that it was especially valuable for them to talk to the other fathers in the fathers’ group. There, the fathers could speak about their issues without the mothers present. MFT led to ‘aha-experiences’, as well as giving structure to chaos. One of the fathers spoke of his loneliness and frustration when outsiders asked how it was going with his daughter. The answer was often that it was not good with her, but those who had inquired had neither the time for, nor the possibility of, fully grasping the situation. It struck a chord with the other fathers when he said, somewhat humorously:

*“I have a standard answer – it depends on how long you have. Do you have five or six hours?”* (Father, group interview 1).

Several of the young women with an ED reported that participation in MFT could be both good and bad. They met others but could also feel exposed as it was apparent to everyone why they were there.

The siblings said that they found it good to meet and talk with other siblings, because there were issues that they did not want to discuss with their parents. It was also helpful to see their parents together with their sister in the MFT. Nonetheless, difficult feelings could be brought up, and participation in MFT good and bad at the same time.

The participants reported themselves as having been treated with respect and having been believed and taken seriously by both the other group members and the therapists. Some said that they had never had such an opportunity as a family, of something so well-tailored to them. The families challenged each other more than the therapists did. Some said that they had almost forgotten that the therapists were there in the MFT, and that what was most beneficial was the conversations with the other young woman and the family members involved in the MFT. Even though the therapists had both expertise and empathy, they nonetheless did not have the same lived understanding of how it is to live with an ED as did the other families in MFT.

*“The therapists know a lot, but still can’t have the same understanding of your problems as do the others who have exactly the same problems. You have the feeling that they don’t quite get what you’re talking about, even though they know a lot about it. It’s, plain and simple, just not possible to understand the pain and anxiety without having been there yourself.”* (Mother, group interview 2).

### Opening up and sharing

The participants reported that being in the MFT had given rise to greater openness.

They experienced their encounter with others with comparable experiences who understood how it was in their family, as beneficial. The meeting with these other families created an underlying safety in the group. They experienced the MFT group as a place tolerant of their need to cry or to be angry. The group dynamic in MFT, and safety in the group, led to the group members opening up and sharing their experiences. The parents became more conscious of the importance of openness in relation to the daughter with an ED, and the young women got to see how things were for their parents.

*“It was help with tidying and sorting - what’s mine, what’s my sister’s, what’s Mum and Dad’s.”* (Young woman, individual interview 2).

In working with young adults with an ED, MFT focuses more on issues around communication and cooperation than directly on the symptoms themselves. Several of the participants talked about being stuck, and about being anxious about triggering the ED and difficult emotions. A father described it as being like a wall between his daughter and the rest of the family.

*“Before we started in MFT it was just as if there was a wall between her and us in relation to the disease, but then something happened in the MFT which meant that the wall was broken down a bit. You knew what we could talk about, have some shared experiences around the illness that we could speak about – because we have been so scared – am I crossing a boundary here if I say …*. *or is it something I mustn’t say. You become very watchful. This wall really set a limit in that she didn’t really know what to say to us, because we didn’t really understand what this was all about. And so, you sit on the other side of the wall, afraid that if you say this or that it might be wrong, or there again maybe smart. You sit and wonder what to do: should you try to tempt her with something? Should she get something if she does it? You sit there like a question mark. And you don’t start to climb over the wall because you don’t know what awaits you on the other side …*” (Father, individual interview 5).

In MFT, the participants are helped to open up about the ED and the difficulties it causes in the family. The aim is to ‘tear down the walls’ which are shutting off the young women with an ED, shutting off family members and the family as a whole and which stand between the families both in- and outside of MFT and friends, network, the health care services and society in general. The “wall” which the father spoke about can be seen as a symbol, picture or metaphor of this closedness. Two of the most important aims that MFT seeks to achieve are greater openness and better communication, both within the family and beyond it.

In the MFT, unhappy patterns could be broken down, and talking to other families and seeing how they handled similar problems found to be helpful. Several of the group members said that it could be easier to talk about difficult matters in the large group with everyone together, than as a family. It was often easier to put difficult questions to the other families. This may be due to the communication in the birth family having become locked and conflictual. They also found it helpful to mix up the families, to find oneself another mother or daughter. It felt good to the young women to meet other parents, and they often dared to ask them questions they had struggled to ask their own. One of the young women said that the MFT led to forgetting herself a little. She could say things aloud in the large group, in front of everyone, that she never would have dared say at home.

In MFT, there were others who understood, and who gave confirmation – looked at you and nodded whilst you spoke, recognised what you were saying. People had experiences complementary to yours, it was like that too. They discussed, other families came with suggestions for solutions, how they do things, what others do that is good or not so good. The participants said that they had learned from the other families. They gained new relational tools and learned to work together in new ways.

Some of the participants in MFT said that the young women talked more, smiled more, showed more of their healthier side and behaved more openly than they usually did at home or at the hospital where the ED often claimed even more space. This may be because the main focus of the MFT for young adults is not the ED, food or illness, but rather communication and cooperation. At the same time, the young women were quiet and shy, and little forthcoming in the group discussions.

*The young women with EDs were asked to write, by themselves, some key words about what their life would be like without an ED. Then they shared between themselves about the words they had written down, and the conversation flowed much more easily. In the plenary group, the therapist improvised: “I have agreed with the young women that we will exploit the good atmosphere of our peer group and share with everyone else in the large group. The other participants can ask the young women concrete questions and they will tell you what we have talked about.” It turned out to be a good concrete conversation, neither philosophical nor theoretical.* (Fieldnote).

This simple exercise of writing down keywords, first for themselves then shared with each other in the young women’s group, led to increased safety and openness. What followed was a friendly but firm challenge from the therapist to share the same in the big group. The concreteness of the task showed itself to be helpful in both the small and large groups. One of the young women spoke of how using Playmobil figures led to openness and insight in the family group:

*“We set them up in such a way that we could see ourselves and the family. Who stood close by and who stood far away, and which way the faces pointed. A light came on for everyone – how we saw things very differently, but also very much the same. I liked that. Lovely to do something other than just sit and talk.”* (Young woman, group interview 4).

There were many difficult topics, and difficult thoughts and feelings that came up in the MFT. The fathers, mothers and the young women all mentioned, however, humour as rescuing when difficult topics and questions were taken up. Otherwise, it could have quickly got really serious. It could have made it [the MFT] really dismal. Humour loosened things up and was experienced as liberating, it created unity and let the participants relax.

*“It’s almost more important to be able to talk to each other, than moving heaven and earth in trying to get well … Try to live a normal life in so far as one can, talk and have a bit of fun together.”* (Mother, individual interview 6).

Several of the participants said, conclusively, that their family had become much better at talking to each other after having been in MFT. They learned to listen to each other, hear what the others were saying, understanding more of their thinking. And they were able to practice speaking about difficult matters and sharing feelings. As a result, they were able to speak much more openly about everything. This gave rise to increased understanding. Many of the group members expressed gratitude at having been able to take part in the MFT and said that MFT would be helpful to all families, even those where there was neither illness nor diagnosis. Several said that MFT had been the best thing in helping the young women and their families to emerge from the ED, and some said that they could well imagine participating in the course several times. Some of the young women said that the MFT had been most important for their parents, but that it helped them indirectly in that things had become better at home, with improved communication.

*“I believe that MFT has been the most important thing in getting well. I learned that it is important to say what you are feeling; learned to speak. When the family understands more, it’s better equipped to be supportive, so that you don’t have to do the work of getting well completely by yourself. It is so good! We wouldn’t have the relationships we have* [in the family*] if it hadn’t been for MFT, guaranteed.”* (Young woman, individual interview 1).

## Discussion

Our findings suggest that MFT for adults with an ED contributed to “Connectedness and recognition”. Feelings of shared interests and safety gave rise to “Opening up and sharing”.

Group members said that there was improved communication and more cooperation in their family after participation in the MFT. Here, our main findings are discussed with reference to earlier research. Finally, the role of MFT in the treatment of adults with an ED is discussed.

Central to MFT is that it brought together several families from different backgrounds but having in common the shared experience of having an adult daughter suffering an ED. They found in it a space within which they could share, and have recognised, the difficulties within their family with others who understood because they had similar difficulties. This created a powerful feeling of ‘being on the same wavelength’ and a fellowship and solidarity both liberating and painful [28].

*Cohesion* is a known phenomenon in group psychotherapy, described in a number of studies of the different types of group therapy [44, 45]. Burlingame and colleagues [45] discuss different forms of cohesion, such as vertical cohesion, horizontal cohesion, task cohesion and affective cohesion. In our study, several of the participants stressed the importance to them of *horizontal cohesion* – their relation to others in the group and to the group as a whole - and of *affective cohesion.*

Studies of MFT with adolescents and adults suffering an ED have produced similar results. Scholz and Asen [16] and Asen and Scholz [18] write that MFT fosters *solidarity*, support, hope and reduced family isolation. Unlike single family therapy, MFT forms a sharing *community* of families with similar difficulties and experiences, and this mutuality has the powerful and supportive effect of *being in the same boat,* relieving some of the burden felt by carers [16]. Those with lived experience are uniquely placed to offer hope and can be more influential and empowering than the messages delivered by health professionals [16]. This corresponds with our finding showing that the families challenged each other even more than did the therapists.

Dawson and colleagues [28] found that MFT fosters social and personal resources by *creating community*, and that the core principles of MFT include *creating solidarity*. Tantillo and colleagues` [22] study showed that parents in MFT found it refreshing to be with others who really understood, experiencing it as being like *coming home.* Voriadaki and colleagues [17] found that open expression of feelings in MFT had a positive effect and promoted *cohesiveness* in the group even where difficult emotions were expressed. Engman-Bredvik and colleagues [46] found that *peer support* was of crucial importance, particularly to the fathers [[46] , p. 194]. One of the fathers in our study made the point that the company of other fathers, being able to speak with each other undisturbed, had had a profound importance. Similarly, Dimitropoulos and colleagues [20] described how patients and their family members had found sharing with others who understood their struggles with AN to be the most beneficial aspect of the MFT.

Encouraging open communication is one of the core values of MFT [18, 28]. This is in conformity with our finding: “Opening up and sharing”. Some of the young women with an ED in our study were unassuming and found it difficult to share themselves with the big group. They often spoke with lowered voices and answered in monosyllables. Withdrawnness and lack of response may be interpreted as the result of executive function impairments such as reduced cognitive flexibility and decision-making capacity caused by the ED [47, 48], and by extreme dietary restriction [49], but can also be understood in terms of shyness, or the need for self-protection [50]. This can be perceived as a manifestation of isolation and inability to communicate.

One of the fathers in the study described there being *a wall* between his daughter and the rest of the family, meaning by this that communication within the family had become closed off and limited. Tantillo and colleagues [22] write about the *disconnection* pointed to by adults with an ED and their family members in MFT. This is a reference to the loss of connection between the patients and their feelings and the disconnection between family members one from another (which may be caused by feelings of guilt or blame about the cause of the ED, and poor emotional bonds [51], or by patients withholding information in order to maintain their autonomy [20]), to the effects of not knowing what best to say, of trying to avoid conflict, of misunderstandings and of sheer exhaustion. Also, disconnection from friends, colleagues, the wider family and the outside world generally [[22], p. 279). This disconnection was symbolised in our study by “*the wall”*, and it appears that receiving help in tearing down *these walls*, in opening up and sharing are the most important things for those who have participated in MFT. This applies equally to the young women with EDs, within their family, between the families and more widely.

MFT provides opportunities to learn from each other in new ways, to establish support and share information about different pathways to recovery. It creates new and multiple perspectives [16] and instills hope [16, 17]. Asen and Scholtz [18] characterise this as a “hothouse effect”. The participants’ increased ability to express emotion was found to be a crucial aspect of MFT. The community, communication and emotional aspects of the therapy and the opening up they led to were beneficial [17, 22]. Engman-Bredvik and colleagues [46] reported that the *sharing of inner thoughts* as well as improved family dynamics (competence and collaboration) were seen as most important for participants/parents in MFT. A main finding of Dawson and colleagues` [28] study was *Sharing of insider knowledge.* MFT helped participants think about things in new ways and feel closer as a family [28].

A number of participants in our study reported that they had found it easier to talk about difficult matters in the big group, than in their own family. They also found that it helped to create surrogate families – that is to say, to find a new daughter or new parent. This can be understood as the externalisation of symptoms, and as a significant impact of MFT [16, 22].

In the MFT, many serious and sad themes are addressed. Many of the participants in our study said that humour was important in lightening the mood of the group. Brinchmann and colleagues [31], in an article on the therapists’ role in MFT, also mentioned this. Humour implies the ability to be playful and have an informal style while retaining a seriousness and earnestness of purpose. Schöpf and colleagues [52] consider humour to be relationship building and relationship protecting. It is, however, essential that it be used with sensitivity.

Our findings, “Connection and recognition” and “Opening up and sharing”, appear to be in agreement with the results of earlier studies, both those into MFT with children and young people with an ED and those into adults suffering an ED [16–18, 20, 22, 28, 46]. The main difference between the two is that the parents of minors hold greater responsibility that the ill person eats, whereas adult sufferers hold that responsibility themselves. In MFT for adults there is greater focus on communication and conflict management within the family than on food and the ED itself [10, 30].

Research indicates that the caregivers of those suffering from an ED experience a greater burden of care than do the caregivers of those suffering a psychosis [53]. Some of the main difficulties experienced by families with either a child or young person, or an adult suffering from an ED are stigmatisation, loneliness and isolation [22]. The problem is not diminished when the sufferer is over 18. Society expects that young adults should take care of themselves [5]. Many young adults with a serious, chronic ED have executive function impairments such as reduced cognitive flexibility and decision-making capacity for their age [47, 48] which may make them more dependent on their families than are their contemporaries. Family members’ quality of life is significantly impaired because they carry a burden occasioned by the difficulties their role entails as the patient becomes socially isolated and more heavily dependent on them [6, 7, 26, 27, 53]. Lofthus and colleagues [6] found that parents of adult family members with EDs also felt shut out and isolated in their encounters with the health services. Because their daughter was adult, they were often referred to the duty of confidentiality [6]. These experiences of loneliness, and of there being little help and support, are reinforced by society in general having little awareness and understanding of EDs [5].

Tantillo and colleagues [22] found that MFT helped, indirectly, with the symptoms of AN. In line with this, several participants in our study reported that the solidarity and connectedness they had experienced in MFT had made an impact on recovery and led to an overall improvement in everyday functioning and lessening of certain symptoms. It had also improved communication within the family. Many found the group environment uniquely suited to their needs. Some of the young women thought that it had served the needs of their parents best, but that this had indirectly benefitted them as things had thereby become better at home, with improved communication and cooperation within the family.

### Strengths and weaknesses

There has been little research into the experience of those (young adult women with an ED and families) participating in the MFT. The strength and creativity of this study lies in it being based on extended (2 years) field observations in two MFT groups for adults, supplemented with both group and individual interviews with the participants. The first author (BSB) who carried out the field research was a participatory observer in the MFT, and became well-acquainted with the participants. This led to both her having a unique insight into the MFT and greater safety and openness among the participants than if the researcher had been a remote figure they had never met. In that the researcher participated as an observer in the group on the group’s own terms and therefore the research was practice-led, group interviews had to be conducted in the natural breaks of the group and within the time limits of those. It was not always easy to come into conversation with the group members, especially, sometimes, with the young women ED sufferers. One might suppose that those who agreed to participate in the group and individual interviews were those best resourced, most verbal and most positive about the MFT, and that therefore less positive experiences are not as well represented. BSB has no clinical experience in this field, nor had any expertise with MFT in advance. This could represent both a strength and a weakness. The strength is that MFT has therefore been studied from without, creating the possibility of new perspectives. One may also suppose that the participants would have been more reluctant to criticise the programme, had the researcher been a therapist in the MFT. A potential weakness can be that important themes may have been either misinterpreted or misunderstood. The second author (SK), however, had clinical experience as a psychologist of working with young adult women with EDs, those closest to them and of family work. Two research assistants with experience themselves of being a patient and carer respectively (and of participation in MFT), and two MFT therapists have contributed to the discussion of, and quality-control of, the findings. This adds to the study’s validity and relevance. The reliability and rigour of the study was secured by scrupulously following clearly set out steps in the process of the analysis.

## Conclusion

The study sought to elicit and describe the experience, of both young adult women suffering from severe EDs and their family members, of having participated in MFT. They described the group therapy as being characterised by connectedness and by recognition. There were others there with similar difficulties and it was possible to speak of one’s own family difficulties. This meeting with others in a similar situation created a valuable underlying safety but was both good and freeing, and painful. It made it possible to share the great loneliness of living in a household dominated by an ED, something that outsiders have difficulty in understanding. The parents related how they had been helped to differentiate between realistic and unrealistic concerns and had learned the important of openness and communication in relation to their daughter. Some of the young women said that they found it, directly, most important for their parents, but thereby, indirectly, important for them in that homelife became much better.

### Implications for practice and further research

In Norway, there is a relatively well-developed health care service for children and young people suffering an ED, and their families. Carers of adults with an ED, however, report there being little or a poor service, particularly for those most involved in caring for the ill person. These families carry a heavy burden of caring and need both support and follow-up in order that they can continue to support their ill, adult family member, and to protect their own quality of life and health [6]. The study shows that participants in MFT (for young adults and families) found MFT valuable and important, uniquely tailored to their needs. This suggests that MFT for adults should be further developed and used in the treatment of adults with EDs and of adults in other therapeutic contexts, perhaps most obviously those involving chronic illnesses or substance misuse. More qualitative research is needed to further elaborate the experiences of participating patients, parents and siblings. Quantitative studies into MFT’s effects on the patient and whether, and to what extent, MFT with adults with an ED reduces the symptoms of the ED in those patients. Quantitative studies on the effect of MFT on the health of family members, quality of life and family functioning are also called for.

## Data Availability

Please contact BSB for data request.

## Abbreviations

AN: Anorexia nervosa
BN: Bulimia nervosa
ED: Eating disorder
GT: Grounded theory
MFT: Multifamily therapy
RESSP: Regional centre for eating disorders
